# Biotransformation of caffeoyl quinic acids from green coffee extracts by *Lactobacillus johnsonii* NCC 533

**DOI:** 10.1186/2191-0855-3-28

**Published:** 2013-05-21

**Authors:** Rachid Bel-Rhlid, Dinesh Thapa, Karin Kraehenbuehl, Carl Erik Hansen, Lutz Fischer

**Affiliations:** 1 Nestec Ltd, Nestlé Research Centre, Vers-chez-les-Blanc, P.O. Box 44, Lausanne 26, 1000, Switzerland; 2 Rowett Institute of Nutrition and Health Gut, Health/Microbial Biochemistry, University of Aberdeen, Greenburn Road, Bucksburn, Aberdeen, AB21 9SB, Scotland; 3 Institute of Food Science and Biotechnology, Faculty of Natural Sciences, University of Hohenheim (Stuttgart), Garbenstr. 25, Stuttgart, D-70593, Germany

**Keywords:** Chlorogenic acid, 4-vinylcatechol, Esterase, Decarboxylase, *L. johnsonii*

## Abstract

The potential of *Lactobacillus johnsonii* NCC 533 to metabolize chlorogenic acids from green coffee extract was investigated. Two enzymes, an esterase and a hydroxycinnamate decarboxylase (HCD), were involved in this biotransformation. The complete hydrolysis of 5-caffeoylquinic acid (5-CQA) into caffeic acid (CA) by *L. johnsonii* esterase occurred during the first 16 h of reaction time. No dihydrocaffeic acid was identified in the reaction mixture. The decarboxylation of CA into 4-vinylcatechol (4-VC) started only when the maximum concentration of CA was reached (10 μmol/ml). CA was completely transformed into 4-VC after 48 h of incubation. No 4-vinylphenol or other derivatives could be identified in the reaction media. In this study we demonstrate the capability of *L. johnsonii* to transform chlorogenic acids from green coffee extract into 4-VC in two steps one pot reaction. Thus, the enzymatic potential of certain lactobacilli might be explored to generate flavor compounds from plant polyphenols.

## Introduction

**Figure 1:**
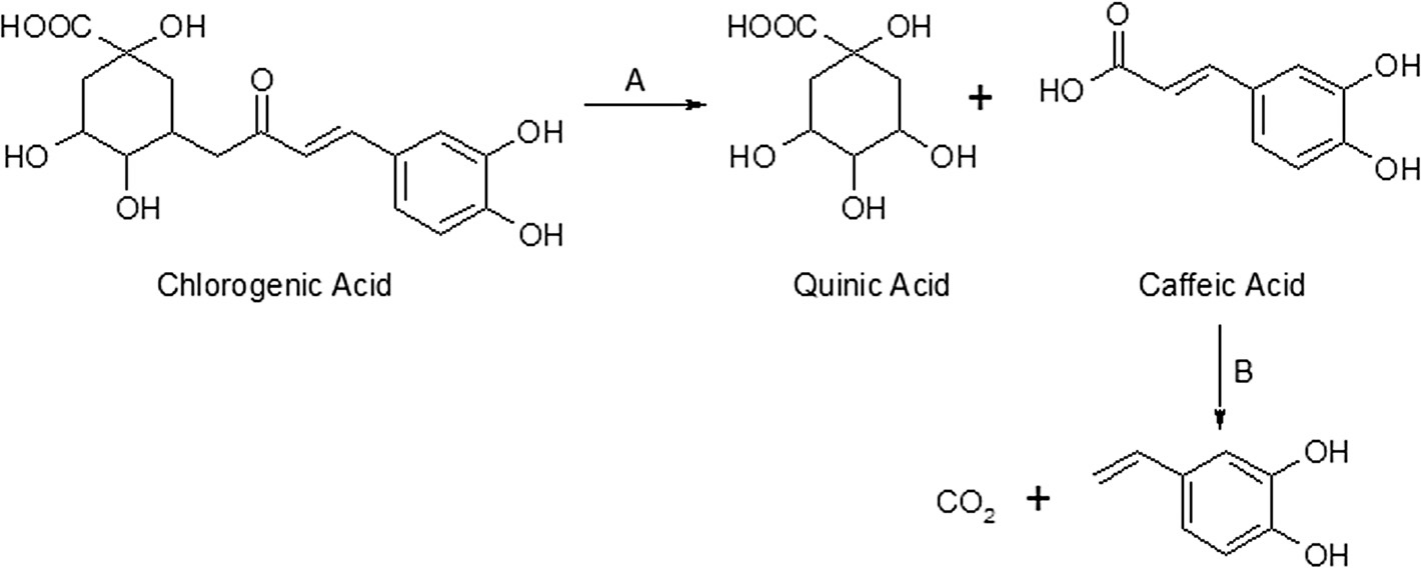
**Biotransformation of chlorogenic acids into 4-vinylcatechol by *L. johnsonii*.** (**A**) cinnamoyl esterase activity, (**B**) hydroxycinnamate decarboxylase activity.

Polyphenols have been reported to exert a variety of biological activities, such as free radical scavenging, metal chelating and modulation of enzyme activity ( Sud’ina 1993 ). The main classes of phenolic compounds are hydroxycinnamic acids such as caffeic acid (CA), ferulic acid (FA), and *p*-coumaric acid (PCA), mainly in esterified form with organic acids, sugars, and lipids. CA (Figure 1) is the major representative of hydroxycinnamic acids and occurs in foods essentially as ester with quinic acid (chlorogenic acids) (Figure 1). Although chlorogenic acids are common in vegetables, the largest amounts are present in coffee ( Clarke 1987 ). These phenolic acids are toxic to some but not all microorganisms. Some *Pseudomonas* strains, as well as *Acinetobacter calcoaceticus*, are able to use them as the sole source of carbon for growth ( Overhage 1999 ). Other studies have confirmed the ability of some lactic acid bacteria to metabolize *p*-coumaric acid into 4-vinylphenol (4-VP) ( Osborn 1997 (van Beek ). One of the mechanisms evolved by microorganisms to counteract phenolic acid toxicity is the induction of enzymes able to metabolize these compounds. Lactic acid bacteria, especially *Lactobacillus plantarum*^2000^ (Barthelmebs ), *Pediococcus pentosaceus*^2000^ (Barghini ) and *Pseudomonas fluorescens*^1998^ ) are able to decarboxylate *p*-coumaric acid and ferulic acid into vinylphenol, vinylguaiacol and vanillic acid. The decarboxylation is catalyzed by a hydroxycinnamate decarboxylase (HCD). This enzyme was found to be produced by different groups of microorganisms including gram negative bacteria, gram positive bacteria and yeasts. Among the gram positive bacteria only lactic acid bacteria ( Rodriguez 2008 ; van Beek 2000 ; Landete 2007 ) and some *Bacillus* species ( Torres y Torres 2001 ; Degrassi 1995 ; Cavin 1998 ; Edlin 1998 ) were identified to display HCD activity. This enzymatic activity might be constitutive or induced when the microorganisms are exposed to exogenous chemicals ( Hashidoko 2001 ). The constitutive expression of HCDs has been reported in yeasts and some gram negative bacteria. The yeasts, *Brettanomyces anomalus* and *B. bruxellensis*, commonly found in wine, are responsible for the production of flavoring compounds such as 4-vinylphenol, 4- vinylguaiacol, 4-ethylphenol and 4-ethylguaiacol ( Edlin 1998 ; Godoy 2008 ; Morata 2013 ) which are the catalytic products of cinnamic acids mediated by HCD. 4-Vinyl derivatives are considered to contribute to the smoky aroma of cured meat products ( Guillén 1998 ). 4-VG and 4-VP have been approved as flavoring agents by regulatory agencies (Joint Expert Committee on Food Additives ( JECFA) 2001 ). 4-Vinyl derivatives have also been reported as potent antioxidants ( Terpinc 2011 ). The HCD activity has been reported in *Lactobacillus* species ( Rodriguez 2008,2008 ; van Beek 2000 ; Landete 2007 ) but not yet in *L. johnsonii*. In a previous study we demonstrated the ability of *L. johnsonii* to hydrolyze rosmarinic acid into caffeic and 3,4-dihydroxyphenyllactic acids ( Bel-Rhlid 2009 ). In the present study, we investigated the *in vitro* incubation of green coffee extract with *L. johnsonii* to hydrolyze chlorogenic acids into corresponding hydroxycinnamic acids. These phenolic compounds could be then transformed into corresponding vinylphenols by decarboxylase activity.

## Materials and methods

### Chemicals

Caffeic acid, *p*-coumaric acid, 5-caffeoylquinic acid (5-CQA) and 4-vinylguaiacol (4-VG) were purchased from Sigma-Aldrich (Switzerland). Dimethyl sulfoxide (DMSO), sodium phosphate (NaPO_4_), acetonitrile (HPLC grade), tri-fluoro acetic acid (TFA) and methanol were purchased from Merck (Germany). Green coffee extract (GCE) was produced from 100% whole beans Robusta coffee (decaffeinated) by counter-current gradient extraction at 110-180°C. The extract was concentrated by evaporation to a solid content of 50% and then spray-dried. GCE was stored as a powder at -20°C until further use.

### Microorganism

Spray-dried culture powder containing *Lactobacillus johnsonii* (NCC 533, 1.19 E10 cfu/g) was kindly provided by Nestlé Culture Collection.

### Incubation of green coffee extract (GCE) with *L. johnsonii* NCC 533

GCE (30 mg) and a spray-dried preparation of *L. johnsonii* (10 mg, 1.19 E10 cfu/g) were suspended in 1 ml phosphate buffer (10 mM, pH 7.0) and incubated at 37°C (Eppendorf, thermomixer) for 24 h. Samples were withdrawn at different reaction times, centrifuged (14000 *g*, 10 min, 4°C), filtered (0.22 μm), and analyzed by HPLC. The influence of temperature, pH, and oxygen on the reaction rate and yield were studied. For all experiments, a reaction control was run in parallel under the same conditions but without bacteria. No formation of caffeic acid and 4-vinylcatechol was observed in all reaction controls.

### Incubation of *p*-coumaric and caffeic acids with *L. johnsonii*

*p*-Coumaric acid (3 μmol/ml) was dissolved in phosphate buffer (50 mM, pH 7.0). To this suspension, a spray-dried preparation of *L. johnsonii* (10 mg/ml, 1.19 E10 cfu/g) was added and the mixture incubated at 37°C for 24 h. Bacteria cells were then harvested by centrifugation, washed with phosphate buffer (10 mM, pH 7.0) and suspended in a solution of caffeic acid (2.75 μmol/ml, phosphate buffer 50 mM, pH 7.0). The reaction mixture was incubated at 37°C for 24 h. Samples were withdrawn at different reaction times, centrifuged (14000 *g*, 10 min, 4°C), filtered (0.22 μm), and analyzed by HPLC for the quantification of CA and 4-VC. Similarly, *L. johnsonii* (10 mg/ml, 1.19 E10 cfu/g) was incubated with CA (2.75 μmol/ml, phosphate buffer 50 mM) at different pHs (5.0, 6.0 and 7.0) for 24 h at 37°C. Samples were withdrawn at different reaction times, centrifuged (14000 *g*, 10 min, 4°C), filtered (0.22 μm), and analyzed by HPLC.

### Liquid chromatography-diode array detection (HPLC-DAD) and liquid

#### Chromatography-mass spectrometry (LC-MS)

Analyses of kinetic study samples were performed on an Agilent-1200 system equipped with a Nucleosil-C18-100-5CC column (4.6 × 250 mm, 5 μm) (Macherey-Nagel, Switzerland) and DAD detector. As 4-VC was not commercially available, 4-vinylguaiacol (4-VG) was used as calibrant without correction factor. Stock solutions were prepared by dissolving separately 5-CQA (20 mg), CA (20 mg) and 4-VG (20 mg) in 10 ml methanol-water solution (70:30, v/v). 250, 500, 1000, 3000 and 5000 μl of these stock solutions were then diluted in 10 ml methanol-water (70:30, v/v) and used as standards for calibration curve. Coffee samples were diluted to 1% solid content and filtered over 0.45 μm pore size syringe filters (Millipore). The column was equilibrated with 50% water containing 0.1% TFA (solvent A) and 50% acetonitrile (solvent B) at a constant flow rate of 1 ml/min and column temperature of 40°C. After injection (10 μl), the gradient elution program was as follows: 0-16 min 12% B isocratic, 16-16.5 min 14.9% B, 16.5-25 min 25% B, 25-30 min 25% B, 30-32 min 100% B and post time 5 min with 12% B. Chlorogenic acids; 3-CQA (rt = 4.55 min), 4-CQA (rt = 7.20 min), 5-CQA (rt = 7.99 min); and CA (rt = 9.97 min) were monitored at 325 nm while 4-VC (rt = 22.35 min.) was monitored at 265 nm.

HPLC-MS was performed on an Agilent-1200 tandem ToF (G6210) system equipped with a XDB C-18 column (4, 6 mm × 50 mm, 1.8 μm). The LC-TOF detector was equipped with an atmospheric pressure chemical ionization source (ESI) operated in negative mode. Experimental parameters were as follows: charging voltage, 2000 V; capillary voltage, 5000 V; vaporizer temperature, 200°C; nebulizer pressure, 50 psig; drying gas flow, 13.0 L/min; gas temperature, 350°C; octopole DC, 35.50 V; fragmentor, 225 V; octopole RFV, 250 V; and skimmer, 60 V. The eluents were 0.1% formic acid in water (A) and 100% methanol (B). The injection volume was 10 μl and a linear gradient from 5% to 67% B in 8 min was used for the separation. 4-VC was detected at a retention time of 3.95 min.

Purification of 4-VC was performed on a Waters preparative HPLC system comprising 2767 sample manager module, 2525 binary gradient pump module and 2996 photodiode array detector module. The separation was achieved on an Xterra preparative column RP18 OBD 5 μm, 19 × 150 mm and guard column RP18 5 μm, 19 × 10 mm. The solvents used were water containing 0.1% v/v of TFA (A) and acetonitrile 100% (B). 1 ml of GCE treated with *L. johnsonii* was injected. The following gradient and flow rate conditions were applied: 0 min 15% B 5 ml/min., 2 min 15% B 20 ml/min., 12 min 25% B 20 ml/min., 13 min 100% B 20 ml/min., then wash with 100% B for 5 min and re-equilibrate at initial conditions for 2 min. Fractions eluting between 8.5-9.2 min were collected, concentrated to 10 ml under slight vacuum (200 mbar) and further evaporated to dryness under high vacuum at a temperature between -60°C and 0°C.

#### Nuclear magnetic resonance (NMR)

NMR data were acquired on a Bruker AVANCE DPX360 NMR spectrometer operating at a frequency of 360.13 MHz for 1H and 90.55 MHz for 13C at 25°C. Samples were prepared in d_6_-dimethyl sulfoxide (DMSO-d_6_, 99.9 atom % D; Sigma-Aldrich). Chemical shifts were measured in parts per million (ppm) values relative to that of the solvent (residual DMSO [H 2.50; C 39.5]). 1D NMR spectra were recorded (7183.91 Hz) with 256 or 512 data points zero filled to 256 or 512. 13C spectra were acquired with 2 K data points zero filled to 16 K. 13C spectra were recorded by using the DEPT pulse with proton pulse at 135 degrees. The spectra were measured under proton decoupling during acquisition and with DEPT polarization transfer pulse sequence. Homonuclear 1H-1H correlation (COSY) was achieved with 1H spectral width between 3033.98 and 7183.91 Hz and using eCOSY sequence with gradient pulses for selection. The data were recorded in 2 K data points in t2 and 128 data points in t1. Data were treated using TopSpin (version 1.3, Bruker GmbH).

δ_H_ (360 MHz; DMSO-d_6_) 4.97 (1 H, dd, *J* 1.2 and 10.9, =CH_2_), 5.45 (1 H, dd, *J* 1.2 and 17.5, =CH), 6.50 (1 H, dd, *J* 10.9 and 17.5, =CH_2_), 6.6-6.7 (2 H, m, ArH), 6.81 (1 H, d, ArH), 8.92 (2 H, br s, OH); δ_C_ (90 MHz; DMSO-d_6_) 110.5, 113.2, 115.7, 118.2, 129.0, 136.9, 145.3, 145.8.

## Results

### Treatment of green coffee extract (GCE) with *L. Johnsonii* NCC 533

**Figure 2:**
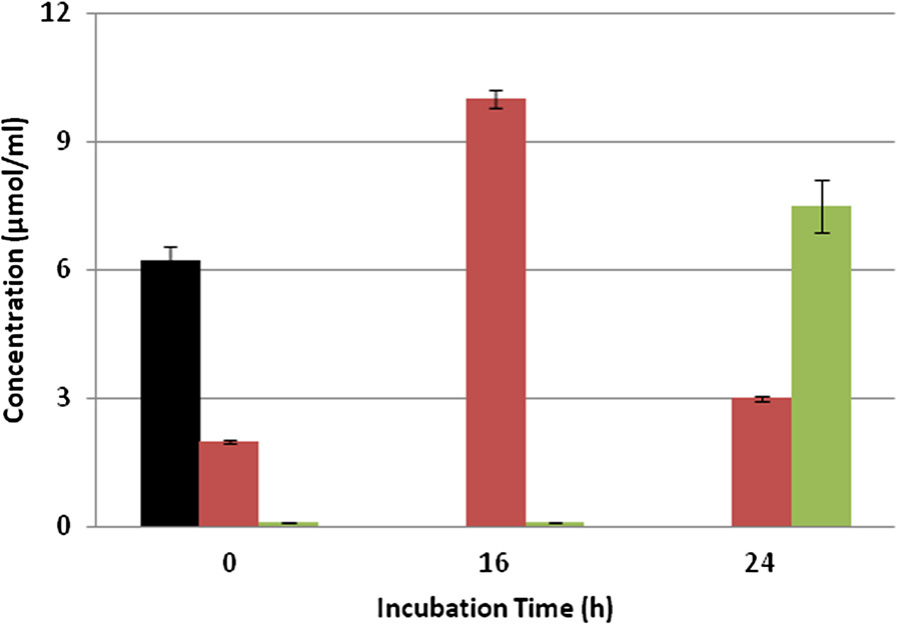
**Bionconversion of chlorogenic acid (5-CQA) (&z.squf;) and formation of caffeic acid (red square symbol) and 4-vinylcatechol (light green square symbol) from green coffee extract by *L. johnsonii *at 37°C.** Values are mean of three different experiments.

GCE was incubated with *L. johnsonii* for 24 h and samples were withdrawn after 0, 16 and 24 h of reaction time and analyzed by HPLC. As shown in Figure 2, during the first 16 h of incubation, the concentration of chlorogenic acids decreased and that of CA increased to reach a maximum of 10 μmol/ml. After 24 h of reaction time, the concentration of CA decreased and 4-VC was produced (7.5 μmol/ml). These results suggest that chlorogenic acids were hydrolyzed by *L. johnsonii* esterase into quinic and caffeic acids and then CA was transformed into 4-VC by a hydroxycinnamic acid decarboxylase (HCD) (Figure 1). No formation of CA and 4-VC was observed in the reaction controls.

### Purification and characterisation of 4-VC

4-VC was purified by preparative RP-HPLC. Upon concentration it tended to undergo quick polymerization. Therefore the final evaporation to dryness was conducted under low temperature and high vacuum. Its chemical structure was elucidated by analytical LC-ToF-MS and NMR (1H, 13C, COSY, DEPT). The molecular mass of 135.061 was measured in the negative mode which was a good match with the mono-isotopic mass of 136.052, expected for 4-VC. 1H and 13C-NMR data were identical to those described in literature ( Nomura 2005 ).

### Reaction kinetics

**Figure 3:**
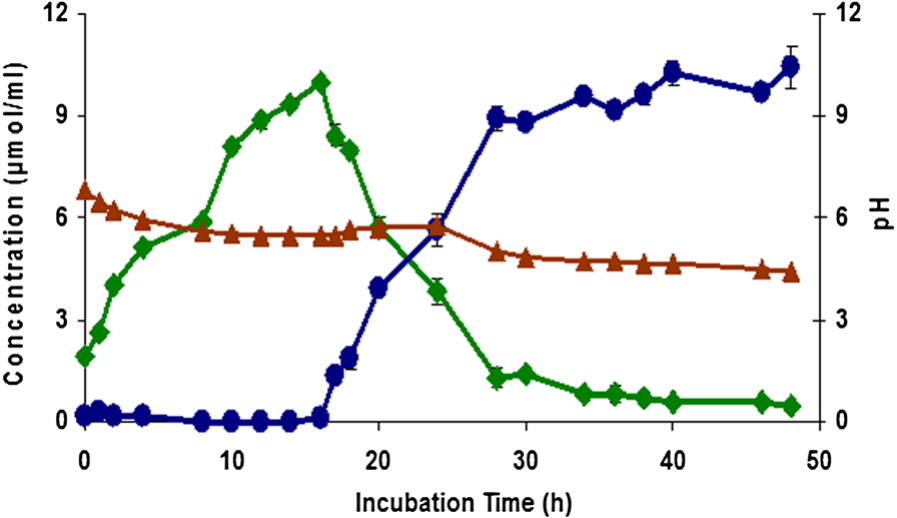
**Kinetics of the generation of caffeic acid (green diamond symbol) and 4-vinylcatechol (blue circle) from green coffee extract by *L. johnsonii *at 37&z.ousco;C.** Variation of pH during reaction (red triangle symbol). Values are mean of three different experiments.

The kinetic behavior of the formation of CA and 4-VC was studied. Reactions were performed in phosphate buffer (10 mM, pH 7.0) at 37°C. As shown in Figure 3, the concentration of CA increased during the first 16 h of incubation and the pH decreased from 7.0 to 6.0. After 16 h of reaction time, 4-VC started to be produced to reach the concentration of 10.4 μmol/ml after 48 h.

### Effect of temperature on the formation of CA and 4-VC

**Figure 4:**
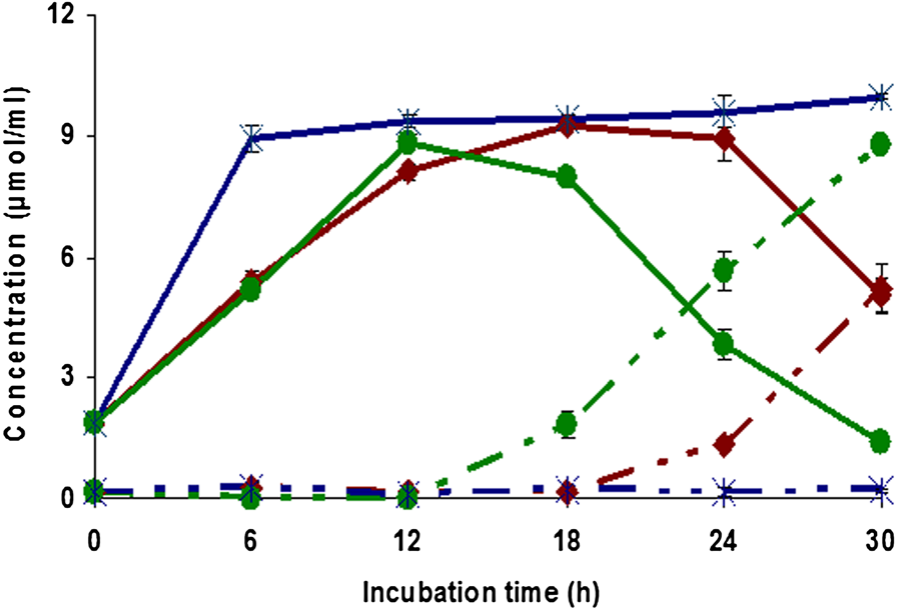
**Effect of temperature on the generation of caffeic acid (solid lines) and 4-VC (dotted lines).** Incubation of *L. johnsonii* with green coffee extract was performed at 30°C (red diamond symbol), 37°C (green circle), and 50°C (×). Values are mean of three different experiments.

The effect of temperature on the formation of CA and 4-VC was studied. Figure 4 shows that the formation of CA and 4-VC was influenced by the temperature. For the CA, no significant difference was observed when the reaction was performed at 30°C or 37°C, whereas at 50°C the reaction rate was much faster. Indeed, at 50°C a concentration of 8.9 μmol/ml of CA was reached after only 6 h of incubation as compared to 5.4 μmol/ml and 5.2 μmol/ml at 30°C and 37°C, respectively. The highest concentration of 4-VC (8.8 μmol/ml) was obtained at 37°C and after 30 h of reaction time, while no 4-VC was produced at 50°C.

### Effect of pH on the formation of CA and 4-VC

**Figure 5:**
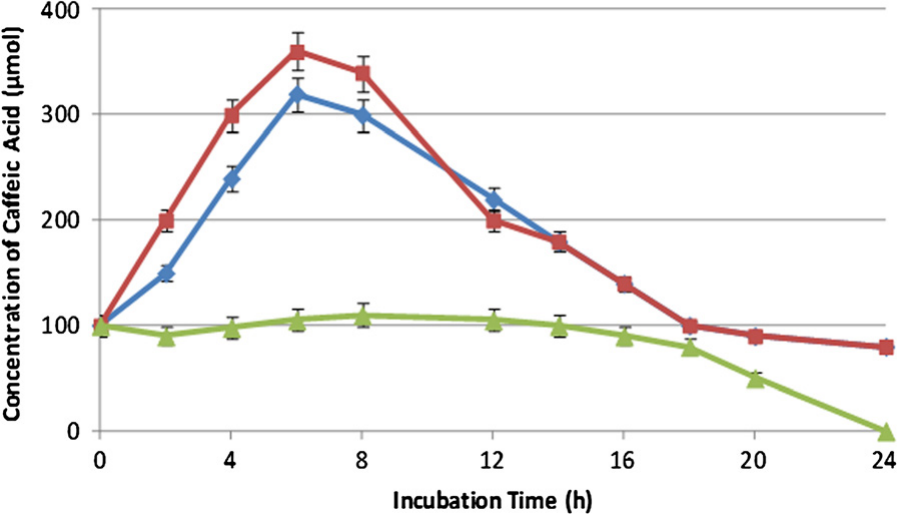
**Effect of pH on the formation of caffeic acid from green coffee extract by *L. johnsonii *at 37°C and pH 5.0 (blue diamond symbol), pH 6.0 (red rectangle symbol) and pH 7.0 (light green triangle symbol).** Values are mean of three different experiments.

**Figure 6:**
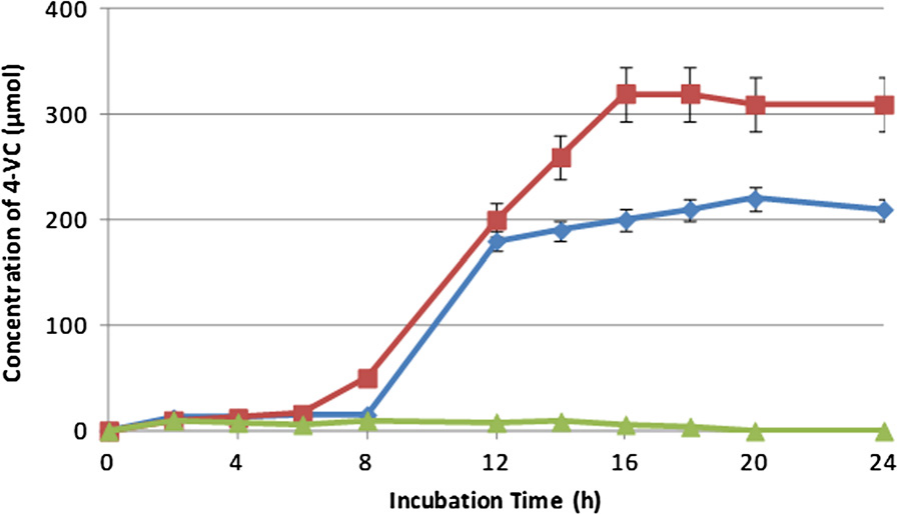
**Effect of pH on the formation of 4-vinylcatechol from green coffee extract by *L. johnsonii *at 37°C and pH 5.0 (blue diamond symbol), pH 6.0 (red rectangle symbol) and pH 7.0 (light green triangle symbol).** Values are mean of three different experiments.

The influence of pH on the formation of CA and 4-VC was studied. Figures 5 and 6 show the formation of CA and 4-VC, respectively, at pH values of 5.0, 6.0, and 7.0. Reactions were carried out using *L. johnsonii* and GCE at 37°C. Constant pH was adjusted by continuous titration with sodium hydroxide (0.1 N). 4-VC was not produced at pH 7.0. The maximum concentration of 4-VC was reached at pH 6.0, while no significant difference was observed at pH 5.0 and 6.0 for the formation of CA.

### Effect of anaerobic conditions on the formation of CA and 4-VC

**Figure 7:**
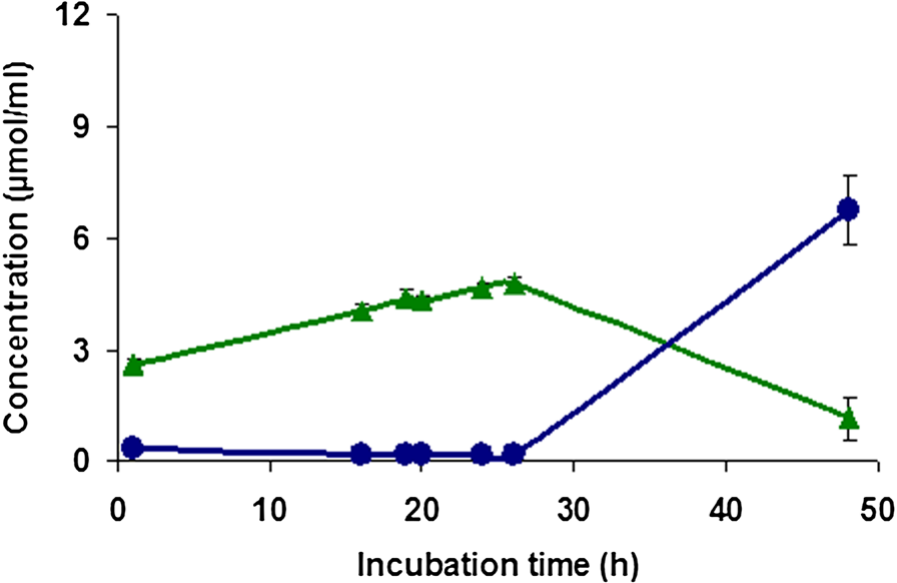
**Formation of caffeic acid (light green triangle symbol) and 4-vinylcatechol (blue circle) from green coffee extract by *L. johnsonii *under anaerobic condition and at 37°C.** Initial pH 7.0 was not controlled during the reaction. Values are mean of three different experiments.

Figure 7 shows the influence of anaerobic conditions (nitrogen) on the generation of CA and 4-VC. As compared to aerobic conditions (Figure 3), anaerobic conditions slowed down the formation rate and yield of CA and 4-VC. Indeed, after 26 h of incubation, only 4.8 μmol/ml of CA was formed under anaerobic conditions as compared to aerobic conditions (8.5 μmol/ml). Similarly, after 48 h of reaction time, only 6.8 μmol/ml of 4-VC was generated under anaerobic conditions which is much lower compared to 10.4 μmol/ml produced under aerobic conditions.

### *p*-coumaric and caffeic acids do not induce HCD activity in *L. johnsonii*

*p*-Coumaric and caffeic acids have been reported to induce the expression of HCD in *Klebsiella oxytoca* JCM1665 ( Hashidoko 2001 ) and in *L. plantarum* 748^T^ ( Rodriguez 2008 ; Cavin 1997 ) at a concentration of 2 mM to 3 mM.

In this study, *L. johnsonii* was incubated with *p*-coumaric acid at a concentration of 3 mM in order to induce the HCD activity. Under these reaction conditions no 4-vinylphenol could be identified in the reaction media even after 48 h of reaction time. Pure caffeic acid was also not transformed into 4-VC under the same reaction conditions. The activity test was performed at three different pHs and in all trials caffeic acid was not transformed by *L. johnsonii* and no 4-VC could be identified in the reaction mixture (data not shown).

## Discussion

Chlorogenenic acids (CQA) are found in wide range of vegetables and fruits and are particularly abundant in green coffee (6 – 10% dry basis) ( Clarke 1987 ). Roasting of green coffee beans reduces the amount of chlorogenic acids (2.5-4.0%) ( Debry 1994 ) and hypothetical chemical degradation pathway has been proposed ( Franck 2007 ; Müller 2007 ). On the other hand, intestinal microorganisms ( Couteau 2001 ; Monteiro 2007 ) and other lactic acid bacteria ( Guglielmetti 2008 ) were shown to be able to transform chlorogenic acids into caffeic and quinic acids by cinnamoyl esterase. Several other studies reported on the decarboxylation of hydroxycinnamic acids (e.g. caffeic acid, ferulic acid) into vinyl phenols by bacteria and yeasts ( Plumb 1999 ; Huang 1994 ; Hashidoko 2001 ; Rodriguez 2008 ; Curiel 2010 ). However, to our knowledge, this is the first study reporting on two step, one pot bioconversion of caffeoyl quinic acids into 4-VC by the same microorganism (Figure 1). Our study reveals that *L. johnsonii* NCC 533 exhibits a chlorogenate esterase and a hydroxycinnamate decarboxylase like activities when incubated with green coffee extract for 24 h at 37°C. The chlorogenate esterase from *L. johnsonii* was already identified, cloned, and characterized ( Bel-Rhlid 2009 ; Kim 2009 ). This enzyme showed high affinity and catalytic efficiency toward aromatic compounds such as chlorogenic acids. HCD has never been reported in *L. johnsonii* NCC 533. To explore both enzymatic activities synergistically, *L. johnsonii* was used as whole cell and it was incubated with GCE in a one pot reaction system. The formation kinetics of CA and 4-VC were studied under different reaction conditions. The first reaction, hydrolysis of chlorogenic acids into CA, started quickly, and the highest concentration of CA was generated after 16 h of incubation. After this level of CA was reached, the second reaction, decarboxylation of CA, was started, and after 28 h of reaction time a plateau was reached with maximum concentration of 4-VC. In the literature, the most frequently observed metabolic pathway of CA is by decarboxylation ( Landete 2007 ; Cavin 1997 ; de las Rivas 2009 ) and further reduction to yield 4-ethylcatechol ( Peppercorn 1971 ; Landete 2007 ). Alternatively, CA can be reduced to dihydrocaffeic acid which is then dehydroxylated into *m*-hydroxyphenylpropionic acid. In our study, neither 4-ethylcatechol nor dihydrocaffeic acid were identified in the reaction mixture. Similar results have been reported for *Lactobacillus brevis* strains ( Curiel 2010 ).

HCD activity was reported to be sensitive to the effects of temperature and pH and this sensitivity depends on the type of microorganisms ( Degrassi 1995 ; Cavin 1998 ; Benito 2009 ). In this study, we observed that both reactions, A and B (Figure 1), were markedly influenced by the temperature and pH. Higher temperatures accelerated the rate of formation of CA. At 50°C, reaction A was completed within 6 h, whereas reaction B was completely inhibited (Figure 4). The optimum temperature and pH for HCD activity was found to be 37°C and 6.0, respectively. Similar observations have been made for *p* (Degrassi -coumarate decarboxylase from *Bacillus pumilus*^1995^ ) where optimum temperature and pH were 37°C, and 5.5, respectively. The decarboxylase activity was lost above 42°C and 100% activity was exhibited at a pH range of 5.0 to 6.0. A broad range of temperature and pH has been reported for *p*-coumarate decarboxylase from *Bacillus subtilis.* High activity was observed in temperature range of 30°C to 55°C and pH range of 4.0 to 6.0.

Although the anaerobic degradation of aromatic compounds is not well explored, few studies have shown that decarboxylation reaction may play important initiating roles in the usage of aromatic compounds under anaerobic conditions ( Krumholz 1986 ; Hsu 1990 ). The biotransformation of CQA into 4-VC by *L. johnsonii* (Degrassi was successfully performed under aerobic and anaerobic conditions. Similar results were observed for *Bacillus pumilus*^1995^ ), where the study demonstrated that *p*-coumarate decarboxylase activity was not oxygen dependent. In the case of *L. johnsonii*, the biotransformation rate of CQA into 4-VC was delayed by anaerobic conditions and the final concentration of 4-VC was only 6.75 μmol/ml as compared to 9 μmol/ml obtained under aerobic conditions. In both cases, CA was completely consumed. This suggests that the decarboxylation of CA stopped due to substrate limitation and not to the influence of oxygen. The HCD activity in our studied conditions relies on the catalytic activity of chlorogenate esterase as well. As a result, less CA was produced in the mixture under anaerobic conditions as compared to aerobic conditions.

*p*-Coumaric and caffeic acids have already been shown to induce the expression of HCD at a concentration of 2 mM to 3 mM in *Klebsiella oxytoca* JCM1665 ( Hashidoko 2001 ) and in *L. plantarum* 748^T^ ( Rodriguez 2008 ; Cavin 1997 ). These chemical inducers were also substrates for HCD activity. To investigate the induction of HCD activity in *L. johnsonii* NCC 533, the bacterium was preincubated with *p*-coumaric and caffeic acids separately and the HCD activity was tested with CA as substrate. The cells did not transform CA at all tested reaction conditions. This leaves the assumption that GCE contains some unknown substance which might regulate the expression of HCD in *L. johnsonii*. Moreover, the cell count of La1 decreased by 10-fold during the first 16 h of reaction and reached a little higher account than the initial one after 24 h. The slow dying off of cells might be due to the high concentration of CA reached after 16 h of reaction time. At this point, the cells commenced detoxifying the toxic effect of CA by transforming this compound into 4-VC and started again to grow slowly. The same observation was reported in *L. plantarum* (Barthelmebs and *P. pentosaceus*^2001^ ). However, further experimental work will be required to confirm these hypotheses and to understand the induction and/or activation mechanism of HCD in *L. johnsonii*. The purification and characterization of this enzyme has to be also explored in the future.

In summary, in this study we showed the ability of *L. johnsonii* to transform caffeoylquinic acid (5-CQA) from green coffee extract into 4-VC. The results obtained indicate that two enzymes, an esterase and a hydroxycinnamic decarboxylase, were involved in this biotransformation. Thus, the enzymatic potential of certain lactobacilli might be explored to produce flavor compounds or to enhance bioactivity of plant phenolic compounds.

## Competing interests

The authors declare that they have no competing interests.
